# Measuring vaccine effects on antibiotic use and antimicrobial resistance in low and middle-income countries: A scoping review of methodological approaches, data sources, metrics, and limitations

**DOI:** 10.1371/journal.pgph.0006106

**Published:** 2026-04-17

**Authors:** Chinwe Iwu-Jaja, Chidozie Declan Iwu, Anelisa Jaca, Charles Shey Wiysonge

**Affiliations:** 1 World Health Organization, Regional Office for Africa, Brazzaville, Congo; 2 Department of Epidemiology, School of Public Health, University of Washington, Seattle, Washington, United States of America; 3 Cochrane South Africa, South African Medical Research Council, Cape Town, South Africa; Aga Khan University, PAKISTAN

## Abstract

Vaccines represent a critical strategy for combating antimicrobial resistance (AMR), yet methodological approaches for assessing their impact remain heterogeneous, particularly in low- and middle-income countries (LMICs) where both infectious disease and resistance burdens are highest. This scoping review systematically mapped the methodological approaches, data sources, and outcome measures used to evaluate vaccine impacts on AMR in LMICs. We searched PubMed, Web of Science, Scopus and CINAHL for studies examining vaccine-AMR relationships in LMICs. Two reviewers independently screened studies and extracted data on study designs, data sources, outcome measures, and limitations. We followed Preferred Reporting Items for Systematic reviews and Meta-Analyses extension for Scoping Reviews guidelines. Sixty-two studies met inclusion criteria, including 58 primary studies and 4 reviews. Among primary studies, 45 were observational (72.6%), 11 were modelling studies (17.7%), 2 randomized trials (3.2%), and 4 systematic reviews (6.5%). Evidence concentrated heavily on pneumococcal conjugate vaccines, with minimal research on other WHO priority pathogens. Data sources included surveillance networks, carriage surveys, national reference laboratories, and administrative immunization records. Primary outcome measures included resistance prevalence (n = 58), serotype replacement (n = 18) and antimicrobial use data (n = 23) such as antibiotic prescriptions or defined daily doses. Common limitations included surveillance biases, incomplete vaccination records, single-site generalizability constraints, and limited linkage between microbiological and clinical outcomes. Substantial methodological heterogeneity characterizes vaccine-AMR research in LMICs, with systematic gaps in antimicrobial use data and evidence beyond pneumococcal vaccines. Strengthening digital health infrastructure, integrating antimicrobial use measurements into existing surveys, embedding resistance endpoints in vaccine trials, and standardizing outcome definitions across studies are essential to generate policy-relevant evidence for immunization programs in high-burden settings.

## Introduction

Antimicrobial resistance (AMR) represents one of the most pressing global health threats of the 21st century, with an estimated 4.71 million deaths linked to bacterial AMR in 2021 including 1.4 million deaths in South Asia and 1 million in sub-Saharan Africa [1]. The burden of AMR disproportionately affects low- and middle-income countries (LMICs), where higher infectious disease burdens, lower vaccine coverage, and increased rates of inappropriate antimicrobial use create ideal conditions for the emergence and spread of resistant pathogens [2].

Vaccines represent a critical but underutilized strategy in the global response to AMR [3]. By preventing infections, vaccines can reduce AMR through multiple interacting pathways: directly preventing drug-resistant infections, preventing viral infections that lead to inappropriate antibiotic use and secondary bacterial complications. reducing overall antimicrobial consumption, and decreasing the selective pressure that drives resistance development [2,4,5]. The World Health Organization (WHO) estimates that existing and new vaccines could prevent up to 515,000 AMR-associated deaths and save $30 billion in hospital costs annually in Africa [5].

Empirical work from LMICs shows that childhood vaccines such as the pneumococcal conjugate vaccine (PCV) and rotavirus vaccine reduce antibiotic-treated illness in young children [6,7], and long-running country surveillance demonstrates sustained benefits following routine PCV introduction [8]. In settings with extensively drug-resistant typhoid, programmatic typhoid conjugate vaccine (TCV) roll-out has shown high effectiveness against culture-confirmed disease, including extensively drug-resistant (XDR) strains [9,10]. With vaccines against additional AMR-priority pathogens advancing through clinical development pipelines, establishing standardized methodological frameworks for measuring vaccine impacts on antimicrobial resistance and use is critical. These frameworks will enable rigorous evaluation and cross-study comparisons as these products approach licensure.

Despite the theoretical and emerging empirical evidence supporting vaccines as AMR interventions, reliably measuring the magnitude and effect of vaccines on antimicrobial resistance remains inherently challenging, particularly in low-resource settings [2]. Multiple interconnected challenges complicate assessment efforts in LMICs. Firstly, LMICs face substantial constraints in antimicrobial susceptibility testing capabilities [11]. Quality assurance challenges, including inconsistent sampling practices and inadequate bacteriology diagnostics, further compromise data validity [12]. Additionally, measuring antimicrobial use at individual and population levels presents unique challenges in LMIC contexts, where self-medication and procurement from unregulated outlets are common, national aggregate data may mask local disparities. Further, linking vaccination status with antimicrobial prescriptions requires sophisticated health information systems that are rarely available in low-resource settings [2,13]. Furthermore, the assessment of vaccine impact on AMR requires consideration of multiple outcome measures - from pathogen-level resistance patterns to population-level antimicrobial consumption - each with distinct methodological requirements and limitations [2].

While narrative reviews and policy documents increasingly recognize vaccines’ potential in fighting AMR, the actual research evidence remains sparse and methodologically heterogeneous [2]. A significant knowledge gap exists regarding standardized methodological approaches for assessing vaccine impact on AMR, particularly in LMIC settings where the burden is highest, but research capacity may be limited.

Furthermore, no comprehensive methodological review has systematically examined the approaches, data sources, and analytical methods employed to assess vaccine impact on AMR specifically in low-resource settings. The objective of this scoping review is to systematically identify, map, and synthesize the methodological approaches used to assess the impact of vaccines on antimicrobial resistance and antibiotic use in LMICs. Specifically, we aim to:

1. Identify and characterize the data sources utilized in vaccine-AMR impact assessments.
2. Examine outcome measures and metrics used to quantify vaccine impact on AMR and antimicrobial/antibiotic use.
3. Analyze the methodological challenges and limitations reported.
4. Identify gaps in current methodological approaches and provide recommendations for future research.

## Methods

### Study design and rationale

We conducted a scoping review following the methodological framework outlined by Arksey and O’Malley [14] and refined by Levac et al [15] and reported according to the Preferred Reporting Items for Systematic Reviews and Meta-Analyses extension for Scoping Reviews (PRISMA-ScR) checklist [16]. A scoping review approach was selected as most appropriate for this research question because we aimed to map the existing methodological landscape rather than synthesize specific intervention effects, identify knowledge gaps in methodological approaches, and clarify concepts around measuring vaccine impact on AMR in diverse LMIC settings [17].

### Search strategy

We developed a comprehensive search strategy in consultation with an information specialist. The search strategy combined three key concept areas: (1) vaccines/vaccination, (2) antimicrobial resistance, and (3) low- and middle-income countries, with specific methodology-related terms. We searched the following databases from inception to 10^th^ September 2025; PubMed, Web of Science Core Collection, Cumulative Index to Nursing and Allied Health Literature (CINAHL) and Scopus (S1 Table). We also searched grey literature including WHO technical reports and policy documents.

The search terms were organized into four conceptual domains and combined using relevant Boolean operators, vaccine-related terms, AMR-related terms, and geographic terms specifically targeting low- and middle-income country contexts. The search strategy was first developed for PubMed then adapted for each database’s specific syntax. Non-English articles were excluded given that translation was not feasible within the study timeline and resources.

### Inclusion and exclusion criteria

Studies were included if they examined methodological approaches for assessing vaccine impact on AMR and use, regardless of the specific vaccine or pathogen studied. Studies were eligible if conducted in, or applicable to, low- and middle-income countries (LMICs) defined according to the World Bank country income classification (low-income, lower-middle-income, and upper-middle-income economies) at the time of study [18]. For descriptive reporting, study countries were grouped into geographic regions (Africa, Asia, Latin America and the Caribbean, Europe, and Oceania) using the United Nations geoscheme (UN M49) classification [19].

We included primary research studies, systematic reviews, methodological papers, and grey literature that focused on human vaccines and provided substantive descriptions of data sources, analytical methods, study designs, or outcome measures for vaccine-AMR assessments. All study designs and publication types were considered eligible to ensure comprehensive coverage of methodological approaches.

Studies were excluded if they focused solely on vaccine efficacy or effectiveness without any consideration of AMR considerations, or if research was conducted exclusively in high-income countries with no apparent relevance to LMIC settings. We then excluded studies that examined antimicrobial resistance without any vaccine-related interventions, as well as case reports, editorials, and opinion pieces that lacked substantive methodological content relevant to the assessment of vaccine impact on AMR.

### Study selection process

Two authors - Chinwe Iwu-Jaja (CIJ) and Chidozie Declan Iwu (CDI) - independently screened titles and abstracts using the predetermined inclusion and exclusion criteria. Full-text articles were retrieved for all potentially relevant studies. The two authors (CIJ and CDI) then independently assessed full-text articles for final inclusion. Disagreements were resolved through discussion, with a third author (Anelisa Jaca [AJ]) consulted when required. We used Rayyan software [20] to manage the screening process and maintain an audit trail of inclusion and exclusion decisions.

### Data extraction

A standardized data extraction form was developed and pilot-tested on a sample of included studies. Two authors (CIJ and CDI) extracted data independently, with discrepancies resolved through discussion. Extracted data included study characteristics such as author, year, country or region, study design and methodology, population and setting characteristics, and vaccine(s) studied. Methodological details captured encompassed the data sources utilized, including surveillance systems, electronic health records, surveys, and laboratory data, as well as outcome measures for AMR assessment such as phenotypic resistance, genotypic markers, and antimicrobial consumption patterns. We also extracted information on analytical approaches and statistical methods employed, study design features distinguishing between observational versus experimental approaches and prospective versus retrospective designs.

Given the focus on low- and middle-income country settings, we specifically extracted information on LMIC-specific considerations, including how studies addressed resource constraints, any local capacity building components incorporated into the research, adaptations of methods for low-resource settings, and quality assurance measures implemented to ensure data validity. Additionally, we captured key findings related to methodological performance, challenges and limitations encountered during implementation, and any recommendations for methodological improvements provided by study authors.

### Data synthesis and analysis

Given the anticipated heterogeneity in methodological approaches and the exploratory nature of this scoping review, we employed a narrative synthesis approach. Data were analysed using thematic analysis techniques to identify patterns and themes across included studies. Our analysis framework consisted of four complementary components that build upon each other to provide a comprehensive understanding of the methodological landscape.

The first component involved a descriptive analysis providing a quantitative summary of study characteristics, geographic distribution according to the United Nations classification [21], and methodological features to establish the scope and breadth of available evidence. This was followed by a thematic analysis focused on identifying recurring themes related to data source utilization and limitations, analytical method preferences and adaptations for LMIC contexts, outcome measure selection and validation approaches, resource and capacity constraints encountered, and quality assurance approaches implemented across different settings.

The third component comprised a systematic gap analysis to identify methodological gaps and areas requiring further development, with particular attention to approaches that may be missing or underrepresented in current research. Finally, we synthesized findings into a conceptual framework for methodological considerations specifically relevant to LMIC settings, drawing together insights from all previous analysis components. Results were presented using tables, figures, and narrative synthesis, with visual mappings of methodological approaches developed to illustrate relationships between different assessment strategies and recommendations formulated for standardized assessment protocols suitable for resource-constrained environments.

As consistent with scoping review methodology, we did not conduct a formal quality assessment or risk of bias evaluation of individual studies [17]. However, we noted study design strengths and limitations of the included studies as part of our analysis.

## Results

### Results of the literature search

**Fig 1:**
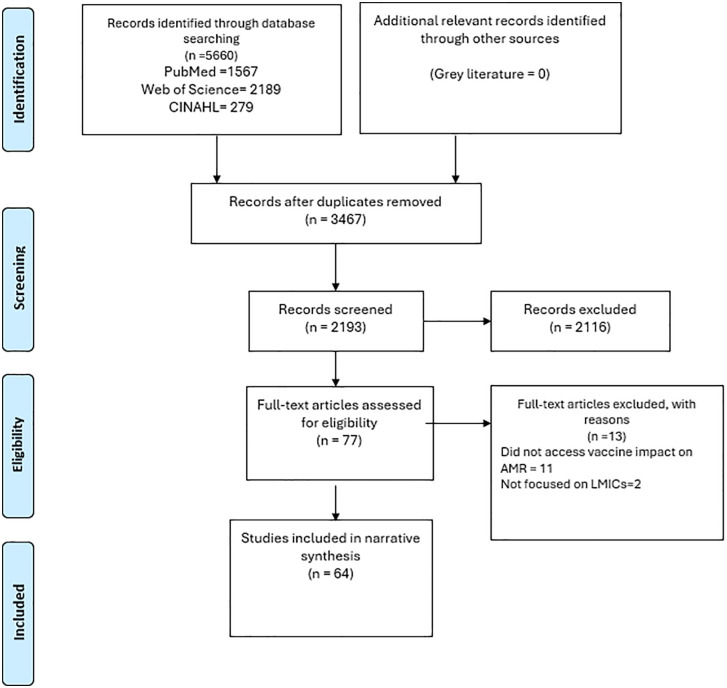
PRISMA diagram of the study selection workflow from identification through final inclusion.

The literature search across three databases PubMed, Web of Science and CINAHL identified 5660 records. Following duplicate removal, 3467 records remained for screening. Title and abstract screening of these records resulted in 2193 records being assessed, with 2116 excluded at this stage. Seventy-seven full-text articles were then evaluated for eligibility. Of these, 13 articles were excluded. Ultimately, 62 studies met the inclusion criteria and were included in the review [9,22–73]. Fig 1 presents the PRISMA diagram illustrating this selection process.

### Characteristics of included studies

The included studies were published between 2012 and 2025. Publication frequency peaked in 2021 with 10 studies, and the majority of all studies (n = 42) were published in the recent period from 2021 to 2025 (S2 Table). A total of nine studies were identified as having a multi-country or global focus that specifically includes or centers on LMICs [22–30].

**Fig 2:**
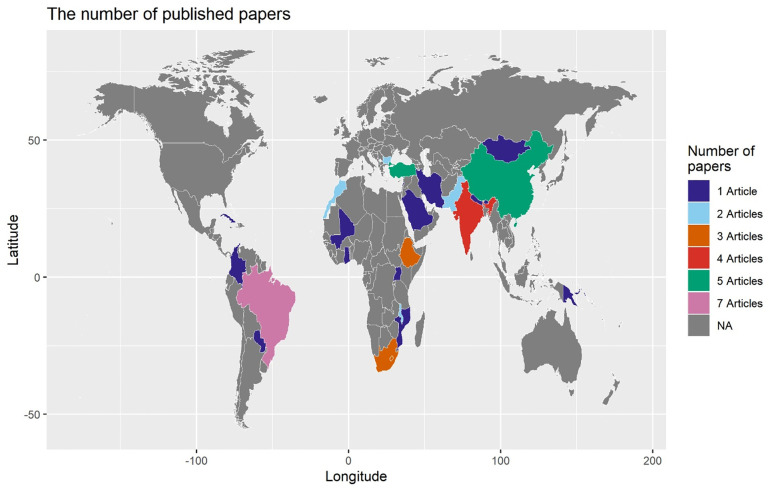
Geographical distribution of included studies (Only single-country studies (n = 52) were included in the map).

We found 17 studies from Asia including China (n = 4) [31–34]; Turkey (n = 4) [35–38]; India (n = 3) [39–41]; Pakistan (n = 2) [9,42] and others, including Nepal [43]*,* Iran [44]*,* Mongolia [45]*,* Palestine [46] and Lebanon [47]. We found 16 studies from sub-Saharan African countries, from South Africa (n = 2) [48,49]; Ethiopia (n = 1) [50]; Morocco (n = 2) [51,52]; Malawi (n = 2) [53,54]; Mali (n = 1) [26]; and others, including Mozambique [55]; Ghana [56]; Botswana [57]; Uganda [58]; The Gambia [26]; Kenya [26]; Burundi [59]; and a multi-country study covering 42 African countries [25]. We found nine studies from Latin America and the Caribbean, with Brazil contributing the majority. Brazil (n = 6) [60–66]. The remaining studies were from Colombia [67], Cuba [68], and Paraguay [69]. We found three studies from Europe and Oceania, including Bulgaria (n = 2) [70,71]; and Papua New Guinea [72]. Fig 2 shows the map of countries and the corresponding studies.

### Study designs

**Fig 3:**
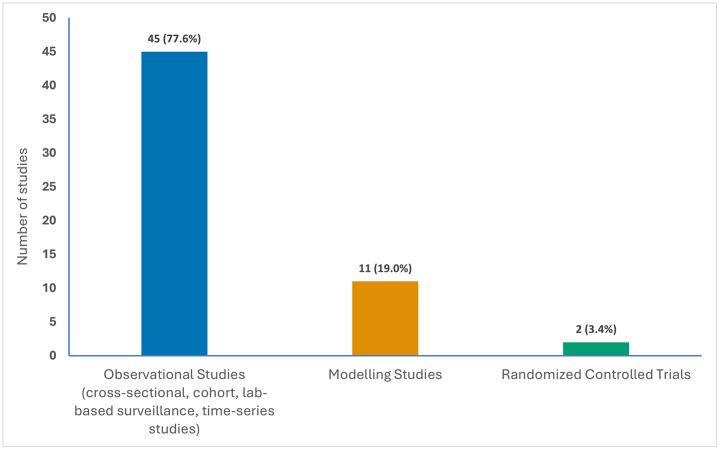
Distribution of study designs among included primary studies*. *Study designs from the reviews were not included in this graph; percentages are calculated among primary studies only (N = 58).

Of the 62 included studies, 58 were primary studies and 4 were reviews. Among the primary studies, the methodological landscape was predominantly characterized by observational designs (n = 45, 72.6%), including cross-sectional studies, prospective and retrospective cohort studies, and laboratory-based surveillance programs. This observational evidence base was supplemented by modelling studies (n = 11, 17.7%), which used mathematical and economic frameworks to simulate and project the long-term health and economic impact of vaccination on AMR dynamics through diverse computational approaches such as agent-based models, deterministic compartmental models, Markov models, and transmission dynamic models. Two primary studies were randomized controlled trials (n = 2, 3.2%) and examined AMR- or antibiotic use–related outcomes as secondary endpoints, including carriage outcomes in vaccine schedule comparisons and antimicrobial prescribing patterns in maternal vaccination trials (Fig 3). cThe six reviews synthesized evidence from observational studies (primarily before-after studies, time series analyses, and surveillance studies), randomized controlled trials, and modelling approaches (see S2 Table).

### Data sources

Studies drew upon diverse data sources spanning clinical surveillance, community surveys, and health system records. Carriage and community surveys were the most common data source (n = 23), utilizing nasopharyngeal and oropharyngeal swabs to assess colonization patterns. Clinical and hospital-based AMR surveillance (n = 18) provided invasive disease data from laboratory antimicrobial susceptibility testing of isolates from sterile sites, including cases of invasive pneumococcal disease, meningitis, and pneumonia admissions. Ten studies utilized national or regional AMR surveillance systems, such as WHO Global Antimicrobial Resistance Surveillance System (GLASS) and country-specific surveillance networks (S2 Table).

Supporting data sources included program and administrative immunization records (n = 10) providing vaccination coverage data from WHO/UNICEF estimates, Demographic and Health Surveys (DHS) surveys, and national Expanded Program on immunization (EPI)reports; genomic sequencing and phylogenetic analyses (n = 5) which employed whole-genome sequencing, multilocus sequence typing (MLST), and molecular serotyping; and model input parameters (n = 3), incorporating pharmacokinetic/pharmacodynamic data, economic cost estimates, and transmission parameters. Two studies accessed randomized controlled trial records, while two others utilized pharmacy and prescribing databases to examine antimicrobial consumption patterns. One study synthesized published systematic review evidence, and eleven studies employed unspecified or other data sources requiring text clarification (S2 Table). Table 1 summarizes these data sources based on the study designs employed.

**Table 1:** Summary of study designs, data sources, outcome measures, and methodological limitations.

Study Design	Primary Data Sources	Primary Outcome Measures	Key Limitations
**Observational Studies**	• **Invasive isolates and surveillance networks:** Isolates from sterile sites (blood, cerebrospinal fluid, pleural fluid) via national surveillance (E.g GERMS-SA in South Africa, Institute Adolfo Lutz in Brazil). • **Carriage/community surveys:** Nasopharyngeal/oropharyngeal swabs from defined cohorts (E.g healthy infants, HIV-infected individuals, hospitalized children). • **Genomic sequencing/phylogenetics:** Whole-genome sequencing and multi-locus sequence typing to track sequence types and clonal complexes	• **Serotype distribution:** Percentage change in vaccine type versus non-vaccine type prevalence in invasive pneumococcal disease and carriage. • **Antimicrobial resistance rates:** Proportion of isolates non-susceptible/resistant to specific antibiotics (penicillin, ceftriaxone, erythromycin, trimethoprim/sulfamethoxazole); multidrug resistance prevalence. • **Incidence rates:** Annual reported rates of invasive pneumococcal disease post-vaccination; incidence rate ratios. • **Risk factors:** Odds ratios for carriage associated with daycare, household size, vaccination status	• **Surveillance biases:** Passive surveillance for non-meningitis invasive pneumococcal disease; high pre-hospital antibiotic use causing negative cultures. • **Incomplete vaccination records:** Difficulty obtaining accurate individual vaccination status. • **Ecological inference limitations:** Single-site data restricts generalizability and causal attribution. • **Confounding by secular trends:** Temporal changes not solely attributable to vaccination
**Modelling Studies**	• **Program/administrative immunization data:** Coverage rates (pneumococcal conjugate vaccine, diphtheria-tetanus-pertussis vaccine as proxy), population demographics from United Nations World Population Prospects. • **Antimicrobial resistance burden and economic inputs:** Baseline estimates from Global Research on Antimicrobial Resistance Project, World Health Organization cost-effectiveness data, local treatment costs and productivity losses. • **Transmission parameters:** Incidence rates, antibiotic utilization rates, pharmacokinetic/pharmacodynamic parameters	• **Cases/deaths averted:** Projected cumulative cases (rifampicin-resistant tuberculosis, malaria, invasive pneumococcal disease) and deaths averted over defined time horizons (2020–2035). • **Economic burden averted:** Cost savings (direct medical costs, productivity losses, hospital costs). • **Resistance dynamics:** Percentage reduction in antimicrobial resistance rates; treatment failures averted. • **Antibiotic use:** Projected reduction in defined daily doses	• **Model assumptions and uncertainty:** Simplified model structures; wide uncertainty intervals due to input variability. • **Parameter heterogeneity:** Extrapolation from specific sites to entire regions (low- and middle-income countries). • **Limited validation data:** Difficulty validating complex dynamics like serotype replacement. • **Generalizability:** Uniform vaccine effectiveness assumptions across diverse contexts
**RCT**	• **Trial/randomized controlled trial records:** Samples from vaccine randomized controlled trials (pneumococcal conjugate vaccine comparison in Papua New Guinea, respiratory syncytial virus F vaccine trial). • **Pharmacy/prescribing data:** Antimicrobial prescription courses during trial follow-up via surveillance or household surveys (Demographic Health Surveys, Multiple Indicator Cluster Surveys)	• **Vaccine effectiveness:** Vaccine effectiveness against new antimicrobial prescription courses (all-cause or syndrome-specific); calculated as vaccine effectiveness = 1 - Hazard Ratio. • **Prescription courses averted:** Absolute number averted per 100 person-years. • **Carriage rates (secondary):** Pneumococcal carriage prevalence (total, vaccine type, antimicrobial resistance) in trial participants	• **Antimicrobial resistance as secondary outcome:** Trials not powered for antimicrobial use or resistance endpoints. • **Limited follow-up:** Restricted observation periods (e.g., 90 days to 27 months) limit long-term assessment. • **Bias in secondary data:** Selection and information biases from mother-reported antibiotic use via household surveys

### Vaccines and pathogens targeted

The research landscape was heavily concentrated on one vaccine-pathogen pair, with pneumococcal conjugate vaccines (PCV7, PCV10, PCV13) dominating the literature (n = 45 studies) and focusing primarily on *Streptococcus pneumoniae* serotype distribution shifts, non-vaccine serotype replacement, and AMR patterns in carriage and invasive disease. Typhoid conjugate vaccine (TCV) targeting *Salmonella typhi* and Paratyphi A comprised a considerably smaller evidence base (n = 3), while other vaccine-pathogen pairs were reported: *Haemophilus influenzae* type b vaccine (n = 2), future tuberculosis vaccines for drug-resistant Mycobacterium tuberculosis (n = 3), malaria vaccine (n = 1), and respiratory syncytial virus F vaccine (n = 1) (S2 Table). The modelling studies explored hypothetical vaccines against WHO priority pathogens, including *Klebsiella pneumoniae*, *Staphylococcus aureus*, *Escherichia coli*, and *Acinetobacter baumannii*.

### Outcomes measures

Vaccine impact on AMR was measured through several direct and indirect outcome measures. Prevalence of resistance was the most common assessed outcome (n = 58), measuring the proportion of isolates resistant or non-susceptible to specific antibiotics, including penicillin, cephalosporins, macrolides, and sulfamethoxazole-trimethoprim, across various pathogen populations. Multidrug resistance (MDR), defined as resistance to three or more antibiotic classes, was examined as a specific endpoint in 24 studies, with particular focus on MDR patterns within specific serotypes and genetic lineages. Serotype replacement served as a key intermediate outcome in 18 studies, tracking shifts from vaccine-type to non-vaccine-type strains as a mechanism underlying changes in resistance prevalence (S2 Table).

Notably, antimicrobial use data were included in only 23 studies, either as a primary outcome (measured through antibiotic treatment courses, prescriptions, or Defined Daily Doses) or as a confounding variable. Primary outcome metrics included reductions in antimicrobial prescription courses**,** defined daily doses (DDDs), and treatment failures averted in modelling analyses (S2 Table).

### Methodological limitations of included studies

The included evidence demonstrated several methodological limitations that varied by study design, with important implications for interpreting vaccine impacts on antimicrobial resistance.

Observational studies are faced with challenges inherent to their design and data sources. The observational nature of these studies prevented definitive causal attribution, with authors explicitly acknowledging that observed resistance changes could not be conclusively linked to vaccination alone due to potential confounding from concurrent interventions, including antibiotic stewardship programmes, public health campaigns, and socioeconomic shifts. Surveillance biases were common due to the passive nature of reporting systems, particularly for non-meningitis invasive pneumococcal disease where surveillance is often non-mandatory. High rates of pre-hospital antibiotic use frequently yielded culture-negative samples, leading to potential underestimation of true disease burden and resistance prevalence. Hospital-based surveillance studies often lacked population representativeness, with samples comprising more severe cases presenting at specific facilities, potentially overestimating resistance rates compared to the general community and limiting generalizability. Incomplete vaccination records, stemming from non-computerized medical archives and reliance on parental recall, further limited accurate individual-level vaccine effectiveness calculations. Small sample sizes for certain subgroups, such as specific serotypes or age categories, reduced statistical power to detect true effects, while residual confounding from unmeasured variables remained despite multivariable adjustment.

Modelling studies addressed some observational limitations through population-level projections but introduced distinct challenges. Authors emphasized that model outputs represented projections based on assumptions rather than empirical facts. These assumptions included transmission dynamics, vaccine efficacy waning patterns, herd immunity thresholds, human behavioural responses, and simplified demographic representations, often resulting in wide uncertainty intervals. Parameter inputs were frequently derived from different sources, requiring extrapolation from single countries or high-income settings to entire low- and middle-income country regions. Validation data for complex dynamics such as serotype replacement or private healthcare sector quality remained limited, reducing confidence in long-term projections and local applicability of uniform vaccine effectiveness assumptions.

Studies utilizing randomized controlled trial data provided higher-quality evidence but with notable constraints. Antimicrobial resistance or antibiotic use were examined as secondary rather than primary trial outcomes, resulting in studies not powered for these specific endpoints and limited statistical power for resistance events. Follow-up durations were restricted (90 days to 27 months), preventing assessment of long-term resistance evolution. Secondary analyses relying on household survey data introduced potential selection and information biases, particularly from mother-reported antibiotic receipt.

Cross-cutting biological and data limitations affected interpretation across study designs. Serotype replacement emerged as the most consistently cited biological limitation in pneumococcal conjugate vaccine studies, with authors noting that reductions in antimicrobial resistance among vaccine serotypes were frequently offset by increases in non-vaccine serotypes, some exhibiting high resistance levels. This ecological effect complicated quantification of net vaccination benefits on overall pneumococcal antimicrobial resistance. A critical gap in antimicrobial use data existed across the evidence base. While modelling studies explicitly quantified reductions in antibiotic consumption as primary outcomes, the majority of observational studies lacked antimicrobial use data entirely, preventing differentiation between vaccine-mediated resistance changes and those driven by antimicrobial selection pressure. Finally, most studies documented changes in microbiological endpoints, such as resistance prevalence, without linking these findings to clinical outcomes (treatment failure, morbidity, mortality) or economic outcomes (healthcare costs), representing a crucial evidence gap between laboratory findings and patient-level impacts.

## Discussion

This scoping review sought to systematically map and synthesize the methodological approaches used to assess vaccine impact on antimicrobial resistance in LMICs. By identifying these approaches, we provide a methodological landscape to guide future research design and investment.

The study designs were comprised of predominantly observational studies, followed by modelling studies, and limited studies utilizing data from RCTs. This methodological distribution reflects both practical realities and inherent challenges in assessing vaccine impacts on AMR. Randomized controlled trials with AMR as a primary endpoint remain scarce due to several fundamental constraints. Ethically, withholding vaccines from control groups to assess resistance outcomes would increase disease risk and antibiotic exposure, making such trials difficult to justify [74]. Practically, AMR is a long-term, population-level phenomenon requiring large sample sizes and extended follow-up periods to detect meaningful changes in resistance rates, making RCTs resource-intensive and often unfeasible [2,75]. Consequently, observational designs dominate the evidence base, not due to methodological inferiority but because they are better suited for tracking AMR patterns across populations and time periods where experimental manipulation is impractical or unethical [76]. Modelling studies have emerged as a critical complement to observational evidence, bridging gaps by simulating long-term vaccination effects on AMR under various assumptions and projecting population-level impacts that cannot be easily measured empirically [5,73]. These models leverage existing epidemiological data to estimate reductions in antibiotic use and resistance, particularly valuable in LMIC settings where comprehensive surveillance data may be limited. However, the limited number of studies utilizing RCT data highlights a missed opportunity: while RCTs remain the gold standard for establishing vaccine efficacy against infection, they infrequently collect antimicrobial use or resistance outcomes as secondary endpoints [74], resulting in underutilization of high-quality experimental platforms for estimating impact of vaccine on AMR.

Given the suitability of observational studies in assessing the impact of vaccines on AMR, these approaches face systematic challenges in LMIC settings, and therefore require context-adapted, phased strategies rather than one-size-fits-all solutions. In many settings, feasibility is constrained by limited laboratory services, inadequate training and workforce, and affordability barriers to diagnostic testing such that families may have to choose between paying for pathogen testing (including susceptibility) and purchasing antibiotics (e.g., neonatal sepsis). Surveillance biases from passive reporting systems, for example, can be addressed through strengthened laboratory infrastructure and harmonized methods and where feasible, sentinel surveillance networks implemented in a phased manner, as recommended by WHO’s GLASS [5,77]. However, in some LMIC contexts the primary constraint is the inability to routinely obtain and process cultures, meaning that culture-based surveillance may be limited regardless of pre-hospital antibiotic use. Where culture is feasible, culture-negative samples resulting from pre-hospital antibiotic use can be mitigated by expanding access to rapid diagnostics and improving specimen collection and transport protocols, while metagenomic sequencing is more appropriately considered a longer-term option given infrastructure, workforce, and cost requirement [4,5]. While incomplete vaccination records may ultimately require strengthened digital health infrastructure, integrated registries, and unique patient identifiers to enable reliable data linkage, incremental approaches may be more feasible in the near term, particularly in paper-based settings (e.g., standardized facility registers, strengthened home-based records, periodic data quality audits, and hybrid paper–digital systems). [4]. Standardizing outcome definitions, harmonizing analytical techniques across sites, and prioritizing evidence generation for high-burden pathogens can improve comparability and robustness in vaccine-AMR impact research.

Modelling studies face distinct challenges related to model structure, parameter uncertainty, and validation, and AMR-inclusive models often require extensive data that are unavailable in many settings, particularly LMICs. Model predictions depend heavily on structural assumptions about transmission dynamics, vaccine efficacy waning, and behavioural responses, with parameter inputs often derived from heterogeneous data sources requiring extrapolation across diverse settings [73,78]. Recent work has proposed a three-pathway conceptual framework (population/pathogen, care, and health outcomes) to incorporate AMR considerations into vaccine economic models, and demonstrates a simplified implementation that leverages available data on antibiotic prescribing and resistance when detailed inputs are lacking [79].To strengthen modelling evidence, researchers should conduct comprehensive sensitivity analyses to characterize parameter uncertainty, use probabilistic approaches to quantify prediction intervals, calibrate models with local surveillance data where available, and where resources permit, employ multi-model comparison frameworks to assess robustness of findings across different structural assumptions [5].

Antimicrobial resistance and antibiotic use are rarely incorporated as primary endpoints in RCTs, resulting in underpowered studies for these specific outcomes and limited follow-up durations [2,78]. This represents a missed opportunity, as recent initiatives demonstrate the feasibility of integrating AMR outcomes into vaccine trials. Some authors have reported that many vaccine trials do not explicitly include AMR as stated outcomes [4] Researchers recommend that Phase III/IV vaccine trials prospectively incorporate antimicrobial use and resistance as secondary outcomes for both primary pathogens and co-infections, with standardized protocols for AMR data collection following established guidelines for antimicrobial susceptibility testing [2,4]. Extended surveillance periods beyond initial efficacy assessments, and where such systems exits, linkage with national antimicrobial consumption databases, and collaboration between vaccine efficacy platforms and AMR surveillance programmes can strengthen this evidence base without requiring standalone AMR-focused RCTs [5,80]. The scarcity of antimicrobial use data represents a fundamental limitation in this review. The majority of observational studies lacked antimicrobial consumption information, preventing differentiation between vaccine-mediated resistance changes and those driven by antibiotic selection pressure [2]. This gap is particularly pronounced in LMICs, where multiple system-level barriers exist: fragmented data collection across paper-based records, limited linkage between prescription databases and surveillance systems, substantial over-the-counter antibiotic access in informal healthcare sectors, and weak laboratory and health information infrastructure [5,77,81]. More broadly, the limited availability of robust data on vaccine impacts on AMR may also reflect that these outcomes are not consistently prioritized in vaccine evaluation and policy decision-making processes; even where evidence exists. Potentialsolutions include integrating standardized antimicrobial use questions into existing household surveys such as DHS and MICS, establishing sentinel site surveillance linking vaccination registries with pharmacy dispensing data, implementing point prevalence surveys of antibiotic use at the facility level following WHO GLASS protocols, and leveraging national antimicrobial consumption reporting systems where they exist [2,5].

Pneumococcal conjugate vaccines were the most studied vaccines, reflecting both the maturity of PCV implementation in LMICs. However, minimal research addressed other high-burden pathogens, including *Salmonella typhi*, *Mycobacterium tuberculosis*, and Gram-negative bacteria such as *Klebsiella pneumoniae* and *Escherichia coli,* for which vaccine candidates remain in early development stages (Frost et al. 2023; World Health Organization 2024) and many others in the WHO’s bacterial priority pathogens list. Expanding vaccine-AMR research to these pathogens presents significant opportunities to apply methodological lessons from pneumococcal vaccine studies, particularly for pathogens where vaccines exist but are underutilized (typhoid conjugate vaccine) or where candidates show promise in clinical trials [82].

This scoping review has several limitations that should be considered when interpreting findings. Our search strategy, while systematic, and conducted in three databases (PubMed, Web of Science, and CINAHL), may have missed relevant studies indexed in regional databases or non-indexed journals common in LMICs. The restriction to English-language publications potentially excluded methodological insights from studies published in other languages. Also, as is typical for scoping reviews, we did not conduct a formal quality appraisal of included studies, which limits the interpretation of the methodological rigor of the approaches described. Our synthesis relied primarily on author-reported limitations and methodological descriptions rather than independent methodological assessment of study execution, meaning we cannot verify whether reported approaches were implemented as described or whether additional unreported limitations existed. Furthermore, we cannot determine whether the limitations reported by study authors reflect actual implementation challenges faced in LMIC settings or merely differences in reporting standards and expectations across journals and research contexts. This distinction is important for understanding which methodological constraints are inherent to LMIC research environments versus which are addressable through improved study design or reporting practices.

## Conclusion

This scoping review systematically mapped the methodological approaches, data sources, and outcome measures used to assess vaccine impacts on antimicrobial resistance in low- and middle-income countries. The evidence base is expectedly to rely predominantly on observational designs drawing from surveillance networks, carriage surveys, and national reference laboratories, supplemented by modelling studies that synthesize diverse epidemiological and economic data sources. Outcome measurement focuses primarily on resistance prevalence and serotype replacement, with antimicrobial use data notably lacking from the majority of studies.

The methodological landscape reveals systematic challenges characteristic of LMIC contexts: passive surveillance limitations, incomplete vaccination records, and fragmented health information systems. However, these constraints need not be insurmountable. Pragmatic solutions include strengthening digital health infrastructure and vaccination registries, integrating antimicrobial use questions into existing household surveys, establishing sentinel surveillance sites with linked laboratory and clinical data, and harmonizing outcome definitions and analytical protocols across studies to enable meaningful comparisons.

Critical evidence gaps persist beyond pneumococcal conjugate vaccines, particularly for WHO priority pathogens where AMR burdens are substantial, but vaccine-AMR research remains minimal. As new vaccines targeting these pathogens advance toward licensure, the findings from this review provide actionable guidance: embed AMR endpoints in vaccine trials from the design phase, prioritize data linkage between microbiological results and patient outcomes, and establish standardized methodological frameworks that balance scientific rigour with feasibility in resource-limited settings. Addressing these methodological imperatives will be essential to generate policy-relevant evidence for immunization programme planning in settings where the dual burden of infectious diseases and antimicrobial resistance is greatest.

## Supporting information

S1 Table Search strategies. (DOCX)

S2 Table Characteristics of included studies. (DOCX)

S1 Checklist PRISMA checklist. From: Tricco AC, Lillie E, Zarin W, O’Brien KK, Colquhoun H, Levac D, et al. PRISMA Extension for Scoping Reviews (PRISMAScR): Checklist and Explanation. Ann Intern Med. 2018;169:467–473. https://doi.org/10.7326/M18-0850.
(DOCX)
