# Supplementary material for: Measuring vaccine effects on antibiotic use and antimicrobial resistance in low and middle-income countries: A scoping review of methodological approaches, data sources, metrics, and limitations
Source: PLOS Glob Public Health. 2026 Apr 17;6(4):e0006106. doi: 10.1371/journal.pgph.0006106 (PMC13089735; doi:10.1371/journal.pgph.0006106)
Supplement: S1 Table — (DOCX) [file pgph.0006106.s001.docx]

**S1 Table: Search strategies**

**PubMed Search History**

Search performed on 10 September 2025

| s/no | Search string | Search results |
| --- | --- | --- |
| #1 | vaccine* OR vaccination* OR immuniz* OR immunis* | 542,349 |
| #2 | "antimicrobial resistance" OR "antibiotic resistance" OR AMR OR "drug resistance" | 20827 |
| #3 | "Developing Countries" OR "sub saharan africa"OR "sub-saharan africa"  OR Africa OR Asia OR "Latin America" OR Caribbean OR "Sub-Saharan Africa" OR "Southern Africa" OR "Eastern Africa" OR "Western Africa" OR "Central Africa" OR "South Asia" OR "Southeast Asia" OR "South East Asia" OR "Central Asia" OR "South America" OR "Central America" OR "Pacific Island*" OR "Middle East" OR "North Africa" OR "low-income and middle-income" OR "low and middle income" OR "BRICS countr*" OR "low income" OR "middle income" OR lmic* OR "developing countr*" OR "low resource" OR Afghanistan OR Albania OR Algeria OR "American Samoa" OR Angola OR Armenia OR Azerbaijan OR Bangladesh OR Belarus OR Belize OR Benin OR Bhutan OR Bolivia OR Bosnia OR Botswana OR Brazil OR Bulgaria OR Burma OR "Burkina Faso" OR Burundi OR "Cabo Verde" OR "Cape Verde" OR Cambodia OR Cameroon OR "Central African Republic" OR Chad OR China OR Colombia OR Comoros OR Comores OR Comoro OR Congo OR "Costa Rica" OR "Cote d Ivoire" OR Cuba OR Djibouti OR Dominica OR "Dominican Republic" OR Ecuador OR Egypt OR "El Salvador" OR "Equatorial Guinea" OR Eritrea OR Ethiopia OR Fiji OR Gabon OR Gambia OR Gaza OR Georgia OR "Georgia Republic" OR Ghana OR Grenada OR Grenadines OR Guatemala OR Guinea OR "Guinea Bissau" OR Guyana OR Haiti OR Herzegovina OR Hercegovina OR Honduras OR India OR Indonesia OR Iran OR Iraq OR "Ivory Coast" OR Jamaica OR Jordan OR Kazakhstan OR Kenya OR Kiribati OR "Democratic People's Republic of Korea" OR Kosovo OR Kyrgyz OR Kirghizia OR Kirghiz OR Kyrgyzstan OR "Lao PDR" OR Laos OR Lebanon OR Lesotho OR Liberia OR Libya OR Macedonia OR Madagascar OR Malawi OR Malay OR Malaya OR Malaysia OR Maldives OR Mali  OR "Marshall Islands" OR Mauritania OR Mauritius OR Mexico OR Micronesia OR Moldova OR Mongolia OR Montenegro OR Morocco OR Mozambique OR Myanmar OR Namibia OR Nepal OR Nicaragua OR Niger OR Nigeria OR Pakistan OR Palau OR "Papua New Guinea" OR Paraguay OR Peru OR Philippines OR Principe OR Romania OR Ruanda OR Rwanda OR Samoa OR "Sao Tome" OR Senegal OR Serbia OR "Sierra Leone" OR "Solomon Islands" OR Somalia OR "South Africa" OR "South Sudan" OR "Sri Lanka" OR "St Lucia" OR "St Vincent" OR Sudan OR Surinam OR Suriname OR Swaziland OR Syria OR "Syrian Arab Republic" OR Tajikistan OR Tadzhikistan OR Tadzhik OR Tanzania OR Thailand OR Timor OR Togo OR Tonga OR Tunisia OR Turkey OR Turkmen OR Turkmenistan OR Tuvalu OR Uganda OR Ukraine OR Uzbek OR Uzbekistan OR Vanuatu OR Venezuela OR Vietnam OR "West Bank" OR Yemen OR Zambia OR Zimbabwe | 1,901,158 |
| #4 | #1 AND #2 AND #3 | 1,567 |

**Web of Science Search History**

Search performed on 10 September 2025

| **s/no** | **Search string** | **Search results** |
| --- | --- | --- |
| #1 | vaccine* OR vaccination* OR immuniz* OR immunis* | 582,504 |
| #2 | "antimicrobial resistance" OR "antibiotic resistance" OR AMR OR "drug resistance" | 269,432 |
| #3 | "Developing Countries" OR "Saharan africa"OR "sub-saharan africa"  OR Africa OR Asia OR "Latin America" OR Caribbean OR "Sub-Saharan Africa" OR "Southern Africa" OR "Eastern Africa" OR "Western Africa" OR "Central Africa" OR "South Asia" OR "Southeast Asia" OR "South East Asia" OR "Central Asia" OR "South America" OR "Central America" OR "Pacific Island*" OR "Middle East" OR "North Africa" OR "low-income and middle-income" OR "low and middle income" OR "BRICS countr*" OR "low income" OR "middle income" OR lmic* OR "developing countr*" OR "low resource" OR Afghanistan OR Albania OR Algeria OR "American Samoa" OR Angola OR Armenia OR Azerbaijan OR Bangladesh OR Belarus OR Belize OR Benin OR Bhutan OR Bolivia OR Bosnia OR Botswana OR Brazil OR Bulgaria OR Burma OR "Burkina Faso" OR Burundi OR "Cabo Verde" OR "Cape Verde" OR Cambodia OR Cameroon OR "Central African Republic" OR Chad OR China OR Colombia OR Comoros OR Comores OR Comoro OR Congo OR "Costa Rica" OR "Cote d Ivoire" OR Cuba OR Djibouti OR Dominica OR "Dominican Republic" OR Ecuador OR Egypt OR "El Salvador" OR "Equatorial Guinea" OR Eritrea OR Ethiopia OR Fiji OR Gabon OR Gambia OR Gaza OR Georgia OR "Georgia Republic" OR Ghana OR Grenada OR Grenadines OR Guatemala OR Guinea OR "Guinea Bissau" OR Guyana OR Haiti OR Herzegovina OR Hercegovina OR Honduras OR India OR Indonesia OR Iran OR Iraq OR "Ivory Coast" OR Jamaica OR Jordan OR Kazakhstan OR Kenya OR Kiribati OR "Democratic People's Republic of Korea" OR Kosovo OR Kyrgyz OR Kirghizia OR Kirghiz OR Kyrgyzstan OR "Lao PDR" OR Laos OR Lebanon OR Lesotho OR Liberia OR Libya OR Macedonia OR Madagascar OR Malawi OR Malay OR Malaya OR Malaysia OR Maldives OR Mali  OR "Marshall Islands" OR Mauritania OR Mauritius OR Mexico OR Micronesia OR Moldova OR Mongolia OR Montenegro OR Morocco OR Mozambique OR Myanmar OR Namibia OR Nepal OR Nicaragua OR Niger OR Nigeria OR Pakistan OR Palau OR "Papua New Guinea" OR Paraguay OR Peru OR Philippines OR Principe OR Romania OR Ruanda OR Rwanda OR Samoa OR "Sao Tome" OR Senegal OR Serbia OR "Sierra Leone" OR "Solomon Islands" OR Somalia OR "South Africa" OR "South Sudan" OR "Sri Lanka" OR "St Lucia" OR "St Vincent" OR Sudan OR Surinam OR Suriname OR Swaziland OR Syria OR "Syrian Arab Republic" OR Tajikistan OR Tadzhikistan OR Tadzhik OR Tanzania OR Thailand OR Timor OR Togo OR Tonga OR Tunisia OR Turkey OR Turkmen OR Turkmenistan OR Tuvalu OR Uganda OR Ukraine OR Uzbek OR Uzbekistan OR Vanuatu OR Venezuela OR Vietnam OR "West Bank" OR Yemen OR Zambia OR Zimbabwe | 4,610,547 |
| #4 | #1 AND #2 AND #3 | 2,189 |

**CINAHL Search History**

Search performed on 11 September 2025

| s/no | Search string | Search results |
| --- | --- | --- |
| #1 | XB vaccine* OR vaccination* OR immuniz* OR immunis* | 83,193 |
| #2 | XB "antimicrobial resistance" OR "antibiotic resistance" OR AMR OR "drug resistance" | 51,360 |
| #3 | "Developing Countries" OR "Saharan africa"OR "sub-saharan africa"  OR Africa OR Asia OR "Latin America" OR Caribbean OR "Sub-Saharan Africa" OR "Southern Africa" OR "Eastern Africa" OR "Western Africa" OR "Central Africa" OR "South Asia" OR "Southeast Asia" OR "South East Asia" OR "Central Asia" OR "South America" OR "Central America" OR "Pacific Island*" OR "Middle East" OR "North Africa" OR "low-income and middle-income" OR "low and middle income" OR "BRICS countr*" OR "low income" OR "middle income" OR lmic* OR "developing countr*" OR "low resource" OR Afghanistan OR Albania OR Algeria OR "American Samoa" OR Angola OR Armenia OR Azerbaijan OR Bangladesh OR Belarus OR Belize OR Benin OR Bhutan OR Bolivia OR Bosnia OR Botswana OR Brazil OR Bulgaria OR Burma OR "Burkina Faso" OR Burundi OR "Cabo Verde" OR "Cape Verde" OR Cambodia OR Cameroon OR "Central African Republic" OR Chad OR China OR Colombia OR Comoros OR Comores OR Comoro OR Congo OR "Costa Rica" OR "Cote d Ivoire" OR Cuba OR Djibouti OR Dominica OR "Dominican Republic" OR Ecuador OR Egypt OR "El Salvador" OR "Equatorial Guinea" OR Eritrea OR Ethiopia OR Fiji OR Gabon OR Gambia OR Gaza OR Georgia OR "Georgia Republic" OR Ghana OR Grenada OR Grenadines OR Guatemala OR Guinea OR "Guinea Bissau" OR Guyana OR Haiti OR Herzegovina OR Hercegovina OR Honduras OR India OR Indonesia OR Iran OR Iraq OR "Ivory Coast" OR Jamaica OR Jordan OR Kazakhstan OR Kenya OR Kiribati OR "Democratic People's Republic of Korea" OR Kosovo OR Kyrgyz OR Kirghizia OR Kirghiz OR Kyrgyzstan OR "Lao PDR" OR Laos OR Lebanon OR Lesotho OR Liberia OR Libya OR Macedonia OR Madagascar OR Malawi OR Malay OR Malaya OR Malaysia OR Maldives OR Mali  OR "Marshall Islands" OR Mauritania OR Mauritius OR Mexico OR Micronesia OR Moldova OR Mongolia OR Montenegro OR Morocco OR Mozambique OR Myanmar OR Namibia OR Nepal OR Nicaragua OR Niger OR Nigeria OR Pakistan OR Palau OR "Papua New Guinea" OR Paraguay OR Peru OR Philippines OR Principe OR Romania OR Ruanda OR Rwanda OR Samoa OR "Sao Tome" OR Senegal OR Serbia OR "Sierra Leone" OR "Solomon Islands" OR Somalia OR "South Africa" OR "South Sudan" OR "Sri Lanka" OR "St Lucia" OR "St Vincent" OR Sudan OR Surinam OR Suriname OR Swaziland OR Syria OR "Syrian Arab Republic" OR Tajikistan OR Tadzhikistan OR Tadzhik OR Tanzania OR Thailand OR Timor OR Togo OR Tonga OR Tunisia OR Turkey OR Turkmen OR Turkmenistan OR Tuvalu OR Uganda OR Ukraine OR Uzbek OR Uzbekistan OR Vanuatu OR Venezuela OR Vietnam OR "West Bank" OR Yemen OR Zambia OR Zimbabwe | 672,632 |
| #4 | #1 AND #2 AND #3 | 279 |

**Scopus Search History**

Search performed on 11 September 2025

| s/no | Search string | Search results |
| --- | --- | --- |
| #1 | TITLE-ABS (vaccine* OR vaccination* OR immuniz* OR immunis*) | 643,575 |
| #2 | TITLE-ABS ("antimicrobial resistance" OR "antibiotic resistance" OR AMR OR "drug resistance") | 239,291 |
| #3 | TITLE-ABS("Developing Countries" OR "Saharan africa"OR "sub-saharan africa"  OR Africa OR Asia OR "Latin America" OR Caribbean OR "Sub-Saharan Africa" OR "Southern Africa" OR "Eastern Africa" OR "Western Africa" OR "Central Africa" OR "South Asia" OR "Southeast Asia" OR "South East Asia" OR "Central Asia" OR "South America" OR "Central America" OR "Pacific Island*" OR "Middle East" OR "North Africa" OR "low-income and middle-income" OR "low and middle income" OR "BRICS countr*" OR "low income" OR "middle income" OR lmic* OR "developing countr*" OR "low resource" OR Afghanistan OR Albania OR Algeria OR "American Samoa" OR Angola OR Armenia OR Azerbaijan OR Bangladesh OR Belarus OR Belize OR Benin OR Bhutan OR Bolivia OR Bosnia OR Botswana OR Brazil OR Bulgaria OR Burma OR "Burkina Faso" OR Burundi OR "Cabo Verde" OR "Cape Verde" OR Cambodia OR Cameroon OR "Central African Republic" OR Chad OR China OR Colombia OR Comoros OR Comores OR Comoro OR Congo OR "Costa Rica" OR "Cote d Ivoire" OR Cuba OR Djibouti OR Dominica OR "Dominican Republic" OR Ecuador OR Egypt OR "El Salvador" OR "Equatorial Guinea" OR Eritrea OR Ethiopia OR Fiji OR Gabon OR Gambia OR Gaza OR Georgia OR "Georgia Republic" OR Ghana OR Grenada OR Grenadines OR Guatemala OR Guinea OR "Guinea Bissau" OR Guyana OR Haiti OR Herzegovina OR Hercegovina OR Honduras OR India OR Indonesia OR Iran OR Iraq OR "Ivory Coast" OR Jamaica OR Jordan OR Kazakhstan OR Kenya OR Kiribati OR "Democratic People's Republic of Korea" OR Kosovo OR Kyrgyz OR Kirghizia OR Kirghiz OR Kyrgyzstan OR "Lao PDR" OR Laos OR Lebanon OR Lesotho OR Liberia OR Libya OR Macedonia OR Madagascar OR Malawi OR Malay OR Malaya OR Malaysia OR Maldives OR Mali  OR "Marshall Islands" OR Mauritania OR Mauritius OR Mexico OR Micronesia OR Moldova OR Mongolia OR Montenegro OR Morocco OR Mozambique OR Myanmar OR Namibia OR Nepal OR Nicaragua OR Niger OR Nigeria OR Pakistan OR Palau OR "Papua New Guinea" OR Paraguay OR Peru OR Philippines OR Principe OR Romania OR Ruanda OR Rwanda OR Samoa OR "Sao Tome" OR Senegal OR Serbia OR "Sierra Leone" OR "Solomon Islands" OR Somalia OR "South Africa" OR "South Sudan" OR "Sri Lanka" OR "St Lucia" OR "St Vincent" OR Sudan OR Surinam OR Suriname OR Swaziland OR Syria OR "Syrian Arab Republic" OR Tajikistan OR Tadzhikistan OR Tadzhik OR Tanzania OR Thailand OR Timor OR Togo OR Tonga OR Tunisia OR Turkey OR Turkmen OR Turkmenistan OR Tuvalu OR Uganda OR Ukraine OR Uzbek OR Uzbekistan OR Vanuatu OR Venezuela OR Vietnam OR "West Bank" OR Yemen OR Zambia OR Zimbabwe) | 6,190,940 |
| #4 | #1 AND #2 AND #3 | 1,625 |
