# Supplementary material for: Measuring vaccine effects on antibiotic use and antimicrobial resistance in low and middle-income countries: A scoping review of methodological approaches, data sources, metrics, and limitations
Source: PLOS Glob Public Health. 2026 Apr 17;6(4):e0006106. doi: 10.1371/journal.pgph.0006106 (PMC13089735; doi:10.1371/journal.pgph.0006106)
Supplement: S2 Table — (DOCX) [file pgph.0006106.s002.docx]

**S2 Table: Characteristics of included studies**

| **Study_ID/**  **Year** | **Country/Region** | **Study design** | **Population** | **Setting** | **Vaccine type** | **Target_AMR_**  **Pathogens** | **Study_**  **Design** | **Primary_Data_Sources** | **AMR_Metrics** | **AMU_**  **Metrics** | **Vaccine_**  **Impact_**  **Measures** | **Limitations (as mentioned by authors)** |
| --- | --- | --- | --- | --- | --- | --- | --- | --- | --- | --- | --- | --- |
| Knupp-Pereira, 2024 | Brazil | Observational, cross-sectional | Pediatric patients (children) | Outpatient/hospital-based setting in Brazil | Pneumococcal conjugate vaccine (PCV10, PCV13) | Streptococcus pneumoniae | Cross-sectional surveillance study of pneumococcal carriage isolates | Laboratory analysis of pneumococcal carriage isolates from nasopharyngeal samples | Resistance prevalence rates; MDR prevalence | NR | Comparison of resistance patterns and serotype distribution before vs after PCV introduction (prevalence change) | Cross-sectional design limits causal inference; single geographic location; carriage isolates may not reflect invasive disease; lack of antimicrobial use data |
| Chikhaoui, 2022 | Morocco (Casablanca) | Observational, retrospective | Hospitalized children (<15 years) with invasive pneumococcal disease (IPD) | Hospital, Casablanca | PCV-13 (introduced 2010), PCV-10 (switched in 2012) | Streptococcus pneumoniae | Descriptive, retrospective review of laboratory-confirmed IPD (2015–2018) | Archived patient records, hospitalization registers, microbiology lab database, death certificates | Resistance prevalence rates (%) | NR | Reported decrease in IPD incidence post-PCV introduction (citing Diawara et al. 2015); serotype distribution: PCV10 (20%), PCV13-nonPCV10 (17.1%), non-vaccine types (17.1%) | Single-center, retrospective design; may not be representative nationally; underestimation of cases; limited serotyped strains restricts conclusions; cannot fully measure vaccination impact in late post-PCV period |
| Ozdemir, 2021 | Turkey (Ankara) | Observational, retrospective | Previously healthy children <18 years with IPD | Single tertiary hospital; lab-confirmed meningitis and sepsis/bacteremia (2009–2019) | PCV7 (introduced 2008), PCV13 (from 2011) | Streptococcus pneumoniae | Descriptive review of IPD cases and isolates; Quellung serotyping; CLSI-based MIC testing | Hospital records; microbiology lab database; CSF/blood culture isolates | Resistance prevalence (penicillin 43.9%, ceftriaxone 9.8% among meningitis isolates); trend over years | NR | IPD incidence in <5y decreased from 9.35/100,000 (2009) to 0.83/100,000 (2019); decline in PCV13 serotypes, rise in non-vaccine serotypes | Single-center; retrospective; non-representative nationally; limited serotyped strains; resistance trends not statistically analyzed across years |
| Lima, 2025 | Brazil (Rio de Janeiro state) | Observational, retrospective descriptive | Children ≤5y (majority) and adults; carriage and IPD serotype 19A isolates (2010–2023) | Public/private healthcare facilities; state surveillance context | PCV10 in NIP since 2010 (high coverage); PCV13 limited (private; limited SUS use after 2019 for risk groups) | Streptococcus pneumoniae (serotype 19A) | Capsular typing (PCR/cpsB), disk diffusion; E-test MICs; MLST (lineages ST733, CC320) | Laboratory isolates from carriage and disease; MLST database | Non-susceptibility rates; MDR proportion; MIC ranges | NR | Shift toward MDR CC320 lineage in late-PCV10 period; context of serotype replacement | Isolate-based; convenience sampling; limited generalizability; small adult sample |
| Farfán-Albarracín, 2022 | Colombia (Bogotá, Cali, Medellín, Cartagena) | Observational, multicenter surveillance | Children <18y with pneumococcal meningitis | 17 hospitals; pre-, transition-, and post-PCV10 periods (2008–2019) | PCV7 (Bogotá 2008), PCV10 nationwide from 2012 | Streptococcus pneumoniae | Ambispective cohort using routine lab methods (Vitek 2/BD Phoenix); Quellung/PCR serotyping; period comparison | Hospital labs; local health departments; National Institute of Health serotyping reports | Resistance proportions across periods; incidence rates | Some prior antibiotic use documented (16%); no systematic consumption metrics | PCV10-type serotypes decreased (75%→9.1%); emergence of 19A (31.8%) and 34 (13.6%); incidence fluctuated with PCR adoption | Heterogeneous periods and sites; vaccination data incomplete for some cases; diagnostic changes (PCR) affected detection |
| Lu, 2021 | China | Modelling (agent-based; DREAMR) | National-level pediatric focus (U5) within model parameters | Country-level simulation of PCV coverage scenarios vs AMR accumulation | PCV (scenarios raising coverage to 85–99%) | Streptococcus pneumoniae (treatment antibiotics: penicillin, amoxicillin, 3rd-gen cephalosporins, meropenem) | Agent-based model linking infections, antibiotic use, PK/PD to AMR; economic module | Published epidemiologic, utilization, cost parameters (national surveys and literature) | Projected % reduction in AMR vs status quo (e.g., penicillin −6.6% scaled; −10.5% accelerated) | Modeled antibiotic utilization by syndrome and setting | Reduced AMR and economic burden with increased PCV coverage (e.g., −$371M to −$586M over 5y) | Model assumptions; parameter uncertainty; China-specific inputs limit external generalizability |
| Lewnard, 2020 | Multiple LMICs (Afghanistan, Angola, Armenia, Burundi, Ethiopia, Haiti, Lao PDR, Malawi, Nepal, Pakistan, Philippines, Senegal, Sierra Leone, South Africa, Tajikistan, Tanzania, Uganda, Zimbabwe) | Observational | Children under 5 years | Community, national household survey data | PCV10/13, Rotavirus | Streptococcus pneumoniae, Rotavirus (antibiotic use outcomes) | Case-control analysis using matched demographic and health survey data | Demographic and Health Surveys (DHS) from multiple LMICs | Incidence of antibiotic-treated ARI and diarrhoea | Episodes of antibiotic treatment | Effectiveness estimates (% reduction): PCV 19.7% reduction in antibiotic-treated ARI (24–59m); Rotavirus 11.4% reduction in antibiotic-treated diarrhoea (0–23m) | Residual confounding possible; vaccine impact estimates uncertain in younger children; reliance on caregiver recall |
| Neves, 2019 | Brazil | Observational | Children <6 years attending clinics (public and private) | Outpatient clinics, Rio de Janeiro | PCV13 (private clinics), PCV10 (national program) | Streptococcus pneumoniae | Cross-sectional carriage study | Nasopharyngeal swabs, lab cultures, questionnaire data | Resistance rates (MICs, disk diffusion) | NR | Low colonization with PCV13 serotypes; observed MDR strains in carriers | Low PCV13 coverage; small sample size; limited representativeness |
| Setchanova, 2017 | Bulgaria | Observational | Children and adults with invasive and non-invasive pneumococcal disease | Hospital and community isolates, national surveillance | PCV10 | Streptococcus pneumoniae | Post-vaccine surveillance study (lab-based) | Clinical isolates from multiple hospitals/labs | Nonsusceptibility rates, MICs, MDR proportions | NR | Decline in vaccine-type IPD; increase in non-vaccine serotypes 19A, 6C | Not all isolates nationally represented; voluntary submission bias |
| Valenciano, 2021 | Mozambique | Observational | Children <5 years, HIV-infected and uninfected | Community and clinic-based carriage surveys | PCV10 | Streptococcus pneumoniae | Cross-sectional carriage surveys (pre- and post-vaccine introduction) | Nasopharyngeal swabs, lab testing | Carriage prevalence, resistance rates | NR | VT carriage dropped after PCV10; penicillin nonsusceptibility declined (HIV-infected from 66%→56%) | VT carriage remained common; no booster/catch-up campaign; regional focus |
| Kandasamy, 2022 | Nepal | Observational | Children (0–14 years) from community, hospital (pneumonia), and sterile-site samples | Kathmandu Valley, hospital and community | PCV10 (introduced 2015) | Streptococcus pneumoniae | Whole-genome sequencing surveillance study | Nasopharyngeal swabs, pneumonia cases, sterile site isolates | Resistance prevalence (before vs after PCV introduction) | NR | Decline in vaccine serotypes; increase in penicillin resistance (15%→32%); highlighted MDR strain GPSC9 | Observational design; genomic data may not capture all drivers |
| von Mollendorf, 2019 | South Africa | Observational | Children <5 years with invasive pneumococcal disease (IPD) | National IPD surveillance, South Africa | PCV13 | Streptococcus pneumoniae | Surveillance-based observational analysis (lab-confirmed IPD cases) | GERMS-SA national surveillance isolates | Resistance rates across vaccine and non-vaccine serotypes | NR | Decline in VT IPD and resistant strains; persistence of MDR non-VT strains | Data may not capture all community cases; limited clinical outcome data |
| Yousafzai, 2021 | Pakistan | Observational | Children <5 years with typhoid fever | Hospital-based blood culture surveillance (Karachi) | Typhoid Conjugate Vaccine (TCV) | Salmonella Typhi | Blood culture-based surveillance study | Hospital laboratories, Karachi | Proportion of XDR and MDR strains in culture-positive cases | NR | Demonstrated TCV effectiveness in reducing XDR typhoid incidence | Limited to specific sentinel sites; short follow-up period |
| Batool, 2021 | Pakistan | Observational | Children 6 months–15 years in peri-urban Karachi | Community and hospital-based matched case-control | Typhoid Conjugate Vaccine (TCV) | Salmonella Typhi | Case-control study (culture-confirmed typhoid vs controls) | Blood culture-confirmed cases, hospital and community controls | Proportion of XDR Typhi among isolates | NR | Vaccine effectiveness: 72% (95% CI: 34–88%) against culture-confirmed typhoid | Short follow-up; outbreak setting may limit generalizability |
| Dayie, 2019 | Ghana | Observational | HIV-infected adults and children | Hospital outpatient clinics, Accra | PCV13 (introduced 2012) | Streptococcus pneumoniae | Cross-sectional carriage study | Nasopharyngeal swabs, lab testing | Prevalence of resistant and MDR pneumococci | Reported cotrimoxazole prophylaxis use | Carriage prevalence 11%; non-vaccine serotypes predominated; 18.5% MDR | Single city; limited sample size; cross-sectional design |
| Patel, 2022 | Botswana | Observational | Children <2 years, with and without pneumonia | Hospital (Gaborone) and community birth cohort | PCV13 | Streptococcus pneumoniae | Prospective cohort studies with molecular serotyping | Nasopharyngeal swabs, molecular PCR serotyping | Carriage prevalence, serotype distribution | NR | Decline in VT carriage; rise in non-VT serotypes 21, 23B linked to resistance | Lacked direct resistance testing for all isolates; limited generalizability beyond setting |
| Cave, 2024 | Malawi | Observational / Genomic surveillance | Children and adults with pneumococcal carriage and invasive disease | Community carriage and hospital surveillance | PCV13 | Streptococcus pneumoniae | Whole-genome sequencing of 1022 isolates (1998–2019) | Global Pneumococcal Sequencing Project; VacSurv Pneumonia study; PCVPA survey | Genotypic resistance markers, SNP analysis | NR | Persistence and clonal expansion of resistant vaccine-type lineages post-PCV13 | Genomic study limited to Malawi isolates; generalizability limited |
| Cassiolato, 2018 | Brazil | Observational / Surveillance | Children <5 and adults with invasive pneumococcal disease | National laboratory-based surveillance | PCV10 | Streptococcus pneumoniae serotype 19A | Surveillance analysis of 9,852 isolates (2005–2017); MLST performed | National laboratory surveillance system (Adolfo Lutz Institute) | MIC values, resistance proportions, MDR definition | NR | Expansion of MDR clonal complex CC320 after PCV10 introduction | Passive surveillance; lack of incidence data; no antimicrobial use data |
| Almeida, 2021 | Brazil | Observational / Genomic surveillance | Children <5 and adults with invasive pneumococcal disease | National laboratory-based surveillance | PCV10 | Streptococcus pneumoniae | Whole-genome sequencing of 466 isolates pre- and post-PCV10 (2008–2013) | National laboratory surveillance isolates, ENA database | Predicted resistance genotypes; MIC thresholds | NR | Observed lineage shifts and AMR trends after vaccine introduction | Subset of isolates analysed; no direct antimicrobial use metrics |
| Kim, 2023 | Global (with regional focus on LMICs: Africa, South-East Asia) | Modelling | Global and regional populations, age-specific groups | Population-level modelling | Existing and hypothetical vaccines (Hib, PCV, TCV, influenza, TB, others) | 15 bacterial pathogens including S. pneumoniae, M. tuberculosis, Salmonella Typhi | Static proportional impact model using GRAM 2019 data | GRAM project data, systematic reviews, surveillance, WHO data | Deaths and DALYs attributable/associated with AMR | Indirect reductions via vaccine-preventable infections | Estimated avertable AMR burden (0.51M deaths; 28M DALYs baseline scenario) | Model-based; assumptions on coverage, efficacy, duration |
| Lu, 2021 | China | Modelling | National-level pediatric focus (U5) within model parameters | Country-level simulation | PCV (scenarios raising coverage to 85–99%) | Streptococcus pneumoniae | Agent-based DREAMR model linking infections, antibiotic use, PK/PD, AMR | Published epidemiologic, utilization, cost parameters (surveys and literature) | Modeled AMR accumulation reductions by antibiotic class | Modeled antibiotic utilization by syndrome and setting | Projected % reduction in AMR vs status quo (e.g., penicillin −6.6% scaled; −10.5% accelerated) | Model assumptions; parameter uncertainty; China-specific inputs limit external generalization |
| Zhai, 2025 | China | Modelling | Adolescents, adults, elderly (age-structured) | National TB epidemic model | Post-infection (PSI, Vaccae-like) and pre/post-infection (PPI, hypothetical) TB vaccines | Mycobacterium tuberculosis (MDR/RR-TB) | Age-, treatment history-, drug resistance-stratified transmission model | Historical demographic & epidemiologic data, WHO TB data | Incidence and mortality of MDR/RR-TB | Indirect (reduction in need for second-line TB treatment) | Elderly PSI vaccination achieved 21% incidence and 18% mortality reduction by 2050 | Model assumptions; vaccine efficacy estimates uncertain; China-only focus limits generalizability |
| Turner, 2025 | Global LMICs | Review | General (children and adults, LMIC focus) | Synthesis across LMIC studies | PCVs, Hib, Rotavirus, Influenza, Typhoid conjugate, RSV (future) | S. pneumoniae, Hib, Salmonella Typhi, influenza-associated bacterial infections | Narrative/scoping review of vaccine impact on AMR | Published studies, surveillance reports, trials | Prevalence and trends of resistant infections | Antibiotic prescription/use data, incidence of febrile illnesses treated with antibiotics | Evidence of reduced antibiotic-resistant disease and reduced prescribing following vaccine introduction | Heterogeneity of data; few studies directly measure vaccine–AMR link |
| Klugman, 2018 | Multicountry (LMIC + HIC examples) | Review | Children, adults (various settings) | Clinical trials and surveillance studies | PCV, Influenza | S. pneumoniae, influenza-associated bacterial infections | Perspective/review synthesizing trial and observational data | South Africa RCT, Finland PCV10 trial, US ABC surveillance, global reports | Proportion resistant isolates; decline in resistant IPD | Antibiotic prescription rates (e.g., PCV7 prevented 35 prescriptions per 100 children) | PCVs virtually eliminated resistant vaccine serotypes; influenza vaccination reduced antibiotic use 13–50% | Not systematic; mixes LMIC and HIC; indirect measures in some cases |
| Gürsoy, 2019 | Turkey | Observational | Children (2 months old, followed longitudinally) | Well-child clinic, tertiary hospital, Turkey | PCV7 | Streptococcus pneumoniae | Prospective cohort before/after vaccination | Nasopharyngeal swabs, lab testing, serotyping | Proportion non-susceptible isolates (%) | Antibiotic use recorded in preceding month | Carriage rate decreased (8.6% → 23%), but penicillin non-susceptibility rose (56.3% → 80.6%) | Small cohort; serotype replacement; context-specific |
| Duarte, 2025 | Brazil | Modelling | National population, age-structured | National-level health system modelling | Pneumococcal conjugate vaccines (PCV10, PCV13) | Streptococcus pneumoniae | Mathematical transmission model with cost-effectiveness analysis | Epidemiologic surveillance data, national health statistics | Incidence of resistant IPD cases; modeled prevalence of resistance | Antibiotic prescribing rates for pneumonia and otitis media | Projected reduction in resistant IPD cases; economic savings | Model assumptions; reliance on national surveillance quality |
| Diawara, 2015 | Mali | Observational | Children under 5 years | Community and hospital, Bamako, Mali | PCV13 | Streptococcus pneumoniae | Carriage surveillance pre- and post-PCV introduction | Nasopharyngeal swabs, lab testing, antibiotic susceptibility assays | Proportion resistant isolates (%) | NR | Decline in vaccine-type resistant pneumococci post-PCV13 | Short-term follow-up; potential biases in community sampling |
| Altun, 2015 | Turkey | Observational | Adults and children with invasive pneumococcal disease | Hospitals in Ankara, Turkey | Baseline for PCV7/PCV13 (pre-introduction data) | Streptococcus pneumoniae | Retrospective analysis of 182 IPD isolates (1996–2008) | Clinical isolates from CSF and blood; susceptibility testing; PCR for macrolide genes | MICs, resistance rates (%) by antibiotic | NR | Baseline AMR and serotype distribution before vaccine roll-out; potential coverage of PCV7/13 | Pre-vaccine study; not assessing direct vaccine impact |
| von Gottberg, 2024 | South Africa | Observational (Cohort surveillance) | All ages (infants to elderly) | National laboratory-based surveillance (GERMS-SA) | PCV7, PCV13 | Streptococcus pneumoniae | National, active surveillance with regression modelling (2005–2019) | GERMS-SA isolates, susceptibility testing, serotyping | Incidence of resistant IPD; proportion resistant isolates | NR | 76% reduction in IPD <2y; significant declines in resistant IPD; indirect adult effects | Non-vaccine serotype replacement in adults; limited to pre-COVID period |
| Hamilton, 2023 | 42 African countries | Modelling | Children under 5 years (hypothetical cohort, 2021–2030) | Continental modelling study | Malaria vaccine (RTS,S/AS01; R21/Matrix-M) | Plasmodium falciparum (drug-resistant strains) | Compartmental transmission model with multiple VE scenarios | WHO malaria data, drug resistance trends, published trials | Incidence of drug-resistant malaria cases; deaths averted | Indirect reduction in antimalarial use | Up to 1 resistant case and 1 death averted per 1000 vaccinated children; greater impact with sustained VE | Model assumptions; uncertainty in vaccine efficacy decay rates |
| Fu, 2021 | 30 high-burden countries (India, China, Indonesia, Pakistan, Nigeria, South Africa, etc.) | Modelling | Adults and adolescents with latent TB infection | National and multicountry modelling (high TB burden LMICs) | M72/AS01E (post-exposure TB vaccine, hypothetical rollout) | Mycobacterium tuberculosis (rifampicin-resistant TB) | Mathematical transmission dynamic model, scenario analysis | Epidemiologic and surveillance data, WHO estimates, published literature | Incidence, mortality, proportion resistant, cases averted | Indirect estimate of reduced empiric antibiotic use for TB-like symptoms | 10–14% RR-TB cases averted; 7.3–31% mortality reduction (depending on scenario) | Assumptions on vaccine efficacy/duration; uncertainty in parameters; limited generalizability |
| Zhou, 2012 | China (Beijing) | Observational laboratory study | Children <5 years with upper respiratory infections | Beijing Children’s Hospital | PCV7, PCV13 (coverage estimates only, not intervention) | Streptococcus pneumoniae | Phenotypic and genotypic analysis of isolates | Nasopharyngeal swabs; laboratory testing (PCR, MLST, serotyping) | Resistance rates, MIC distributions, presence of resistance genes | NR | Estimated vaccine serotype coverage: PCV7 (45.2%), PCV13 (62.2%) | Single center; non-invasive isolates only; cross-sectional snapshot |
| Diawara, 2017 | Morocco (Casablanca) | Observational laboratory and epidemiological study | Children and adults with invasive pneumococcal disease (2007–2014) | Ibn Rochd University Hospital, Casablanca | PCV13 (introduced 2010), replaced by PCV10 (2012) | Streptococcus pneumoniae | Molecular typing (PFGE, pbp genotyping) pre- and post-vaccine introduction | Hospital microbiology lab isolates, invasive samples | Prevalence of PNSP; genotypic profiles of pbp genes | NR | PNSP decreased post-vaccine (31% → 13%); shift in serotypes | Hospital-based; limited generalizability; serotype replacement noted |
| Alexandrova, 2019 | Bulgaria | Observational laboratory study | Patients (0–84 years), invasive and non-invasive isolates (2011–2019) | National surveillance across Bulgaria (Sofia, Plovdiv, Pleven) | PCV10 (introduced 2010, high coverage), PCV13 (contextual) | Streptococcus pneumoniae (serogroup 6 isolates) | Phenotypic (MIC testing), genotypic (PCR, MLST) analysis | Clinical isolates from hospitals and labs | Resistance prevalence to penicillin, ceftriaxone, erythromycin, clindamycin | NR | Emergence of serotype 6C post-PCV10; decline of serotype 6B | Focused on serogroup 6 only; observational design |
| Nshimiyimana, 2013 | Uganda (Kampala) | Cross-sectional observational study | Children 6–60 months (n=194), mostly vaccinated with PCV10 | Mulago Assessment Centre, Kampala | PCV10 (introduced 2013, routine EPI) | Streptococcus pneumoniae, Haemophilus influenzae, Moraxella catarrhalis, Staphylococcus aureus | Carriage prevalence and antimicrobial susceptibility testing | Nasopharyngeal swabs, culture, susceptibility tests | Resistance prevalence per species; MDR proportions | Association with recent antibiotic use (self-reported) | Carriage shifts: ↓ pneumococcus, ↑ H. influenzae; resistant pneumococci persisted | Cross-sectional; limited to urban site; no longitudinal follow-up |
| Paramaiswari, 2025 | India | Observational | Children <5 years with IPD | Hospital and lab-based surveillance | PCV10 | Streptococcus pneumoniae | Cross-sectional surveillance | Hospital isolates, national surveillance | MIC, resistance rates | NR | Reduced resistant serotypes post-PCV10 introduction | Limited to hospital cases, may not reflect community burden |
| Du, 2024 | China | Modelling | National-level pediatric population (U5) | National modelling study | PCV13 scenarios | S. pneumoniae | Dynamic transmission and resistance model | Published epidemiologic and resistance data | Resistance prevalence projections | Antibiotic use reductions modeled | % reduction in AMR under higher PCV13 coverage scenarios | Model assumptions; parameter uncertainty |
| Ashrafian, 2025 | Iran | Observational | Children 18–59 months | Community, multi-center | PCV13 (private sector); PCV10 (national 2024) | S. pneumoniae | Cross-sectional observational study | Nasopharyngeal swabs, PCR, MIC testing | Resistance prevalence | NR | No penicillin resistance in PCV13-vaccinated children; resistant isolates in unvaccinated | Private vaccine access limits generalizability |
| von Mollendorf, 2024 | Mongolia | Observational | Hospitalised children 2–59 months with pneumonia | Hospital surveillance, Ulaanbaatar | PCV13 | S. pneumoniae | Active surveillance with molecular serotyping & AMR gene detection | Hospital surveillance samples | Prevalence of AMR genes, resistance patterns | NR | 43.6% reduction in vaccine-type carriage; reduced AMR gene prevalence post-PCV13 | Hospital-only population; residual circulation of some vaccine serotypes |
| Orami, 2023 | Papua New Guinea | Experimental | Infants (N=262, enrolled at 28–35 days, followed up to 24 months) | Community-based randomized trial, Eastern Highlands Province (PNG) | PCV10 vs PCV13, with/without PPV booster at 9 months | Streptococcus pneumoniae | Open randomized controlled trial (PCV10 vs PCV13, booster PPV or no PPV) with nasopharyngeal swabs at multiple timepoints (1, 4, 9, 10, 23, 24 months) | Nasopharyngeal swabs, bacterial culture, Quellung serotyping, Kirby-Bauer disc diffusion, E-test MIC | Non-susceptibility by disc diffusion and MIC (proportion of isolates resistant or intermediate to each antibiotic); MIC thresholds per CLSI guidelines | NR (study did not measure antibiotic consumption, only resistance) | Comparison of carriage and resistance patterns between PCV10 and PCV13 recipients; prevalence of resistant isolates across vaccine vs non-vaccine serotypes | No unvaccinated control group; carriage isolates may not reflect invasive disease strains; disc diffusion alone may overestimate non-susceptibility; low PCV13 uptake in wider community |
| Arvas, 2017 | Turkey | Observational | Children 0–6 years (n=150) | Well-baby outpatient clinic, Istanbul University Cerrahpaşa School of Medicine | PCV7, PCV13 | Streptococcus pneumoniae | Cross-sectional | Nasopharyngeal swabs, lab cultures, EUCAST 2015 testing | Carriage prevalence; MICs; resistance classification (EUCAST) | Parental-reported antibiotic usage history (not statistically significant predictor) | Carriage rates: 21.4% (PCV7), 11.7% (PCV13 3-dose), 14.4% (PCV13 3+1) | No unvaccinated control group due to high coverage; small sample size |
| Negash, 2019 | Ethiopia | Observational | Children 0–15 years with acute otitis media and SPTM (n=55) | Outpatient pediatric services (3 hospitals, Addis Ababa) | PCV10 | Streptococcus pneumoniae, Staphylococcus aureus, Streptococcus pyogenes, Haemophilus influenzae | Prospective observational | Middle ear fluid cultures, antimicrobial susceptibility testing, cpsB sequencing, Quellung reaction | Resistance proportions (%) by antibiotic | NR | PCV10 introduction led to dominance of serotype 19A pneumococci (73.3%) | Small sample size; hospital-based; limited to children with SPTM |
| Javaid, 2022 | South Africa | Observational / Genomic | Children and household members (≤2 years index children, older family members), Soweto and Agincourt | Community (urban and rural South Africa) | PCV7, PCV13 | Streptococcus pneumoniae | Cross-sectional carriage surveys with WGS | Nasopharyngeal swabs; WGS data; Global Pneumococcal Sequencing project | Resistance rates, MICs, MDR definition (≥3 antibiotic classes) | NR | Decrease in VT serotypes; increase in NVTs (e.g. 11A, 15A, 16F, 34, 35B); decrease in resistance overall but increase in penicillin resistance in NVTs (ages 3–5 years) | Not all isolates sequenced; genomic predictions rely on WGS rather than phenotypic testing |
| da Silva, 2023 | India (Navi Mumbai) | Observational / Genomic | Children with suspected enteric fever; S. Typhi and S. Paratyphi A isolates | Community hospitals and private labs in Navi Mumbai | Typhoid conjugate vaccine (TCV) | Salmonella Typhi, Salmonella Paratyphi A | Prospective genomic surveillance (2018–2021) | Whole-genome sequencing, blood cultures, Bayesian models of spread | Resistance prevalence by antibiotic; genetic clustering | NR | Non-significant reduction in clustering in vaccinated vs non-vaccinated communities; vaccine introduced in phased manner | Vaccination rollout incomplete (COVID delays); observational limits causal inference |
| Peela, 2024 | India | Observational | Patients with pneumococcal isolates (1 month–82 years) | Tertiary care hospitals (Puducherry, Bangalore) | PCV13, Pneumosil | Streptococcus pneumoniae | Descriptive observational; pre- vs post-vaccine introduction comparison | Clinical specimens, hospital microbiology labs | Non-susceptibility rates, MIC values, MDR proportions | NR | Changes in serotype prevalence and resistance rates pre/post vaccine | Single-center data, short post-vaccine window |
| Toledo, 2017 | Cuba | Observational | Children 2–18 months | Community (Cienfuegos municipality) | PCV7-TT candidate (under development, not yet introduced) | Streptococcus pneumoniae | Cross-sectional prevalence study | Nasopharyngeal swabs, lab culture and serotyping | Resistance rates (%) by isolate | NR | Baseline data before PCV introduction | Single municipality, short study period |
| Kasumba, 2023 | The Gambia; Mali; Kenya | Observational | Children 0–59 months with moderate-to-severe diarrhea and controls | Community/sentinel health centers | Rotavirus vaccine (VIDA study context) | Non-typhoidal Salmonella (NTS) | Prospective case-control study | Stool cultures, qPCR, lab testing across sites | Resistance proportions by serovar and site | NR | Trends in NTS prevalence/resistance after rotavirus vaccine introduction | Few isolates in Mali; variability between sites |
| Fortuna, 2023 | Brazil | Observational | Children <6 years old | Community/pediatric clinics in Niterói, Brazil | PCV10 (long-term use, since 2010) | Streptococcus pneumoniae | Cross-sectional carriage study | Nasopharyngeal swabs, culture, PCR, MIC testing | Resistance prevalence, MIC ranges, genotypic markers | NR | PCV10 nearly eliminated PCV10 serotypes; MDR persisted | Restricted to one city; low PCV13 coverage |
| Lewnard, 2022 | Multicountry (including LMICs: Bangladesh, Mexico, Philippines, South Africa) | Experimental | Infants 0–3 months born to vaccinated mothers | Multicountry RCT across 11 countries | Maternal RSV F vaccine (candidate) | Indirect (RSV-related infections, AMR linked via antibiotic prescriptions) | Double-blind randomized controlled trial | Trial records, prescription data | Rates of antibiotic prescriptions (per 100 person-years) | Antimicrobial prescription courses | 12.9% reduction in antibiotic prescribing; prevented 5.1 courses/100 infants in LMICs | Vaccine not licensed; trial context only |
| Nzoyikorera, 2023 | Burundi | Observational | Children <5 years hospitalized with severe pneumonia | Hospital (CHUK, Bujumbura) | PCV13 | Streptococcus pneumoniae | Cross-sectional hospital-based study | Nasopharyngeal swabs, lab cultures, AST | Resistance rates (%) | NR | Decline in VT serotypes post-PCV introduction; persistence of resistant non-VT strains | Single hospital, limited sample size, lack of longitudinal follow-up |
| Matran, 2024 | Multicountry (LMIC focus: Philippines, Morocco, Peru) | Review | NR | NR | Various bacterial vaccines (PCV, Hib, TCV) | Multiple (S. pneumoniae, Salmonella Typhi, Hib) | Narrative review | Published literature | NR | NR | Summarizes evidence that vaccines reduce AMR burden through decreased infections and antibiotic use | Evidence gaps in LMICs; reliance on limited published data |
| Russomando, 2025 | Paraguay | Observational | Children <5 years and adults in same households | Community, hospital-based recruitment | PCV10, PCV13 | Streptococcus pneumoniae | Descriptive cross-sectional carriage study | Nasopharyngeal samples, lab cultures, AST | Resistance prevalence (%) | NR | Persistence of VT serotypes despite vaccination; increase in NVT; MDR strains linked to serotypes 19F, 19A | Limited intrafamilial sample, feasibility and cost barriers for carriage studies |
| Naylor, 2025 | Global (includes LMIC regions) | Modelling | NR | NR | Multiple (including PCV, Hib, E. coli, Klebsiella, S. aureus vaccines) | 14 bacterial pathogens (focus on S. aureus, E. coli, Klebsiella, TB, etc.) | Economic modelling study | Meta-analyses, systematic reviews, hospital cost data, WHO/World Bank datasets | Hospital cost per case, resistance burden | Estimated antibiotic consumption | Projected $207B hospital costs and $76B productivity losses avertable by vaccines (30–40%) | Data gaps for LICs, reliance on secondary data, assumptions in modelling |
| Soysal, 2016 | Turkey | Observational | Healthy children 0–18 years | Community, pediatric outpatient clinics | PCV7, PCV13 | Streptococcus pneumoniae | Prospective surveillance | Nasopharyngeal swabs, lab MIC testing, multiplex PCR serotyping | Resistance prevalence (%) | NR | Lower odds of VT colonization among vaccinated; persistence of NVT serotypes | Regional focus, limited to Istanbul, possible selection bias |
| Al-Lahham, 2018 | Palestine | Observational | Children <5 years with pneumococcal carriage | Community and clinical isolates | PCV7, PCV10, PCV13 | Streptococcus pneumoniae | Cross-sectional surveillance of carriage isolates | Nasopharyngeal swabs and laboratory testing | Resistance prevalence (%) | NR | Decline in vaccine-type resistant strains, increase in NVT | Limited sample size; single-country study |
| Reyburn, 2023 | Multicountry (LMICs included) | Review | All ages (IPD, OM, NPC samples) | Post-licensure observational studies | PCV10, PCV13 | Streptococcus pneumoniae | Systematic literature review of 31 studies | Published studies (MEDLINE, Embase, Cochrane, PubMed) | Resistance prevalence, incidence rates | WHO DDD data (country-level) | Mixed—declines in some studies, increases in others | Heterogeneity across studies, variation in pressure towards AMR |
| Reslan, 2022 | Lebanon | Observational | Children and adults with IPD | 79 hospitals nationwide | PCV7, PCV10, PCV13 | Streptococcus pneumoniae | Nationwide hospital-based surveillance (2005–2020) | Lebanese Inter-Hospital Pneumococcal Surveillance Program | Resistance prevalence (%) | NR | Decrease in AMR but mortality increased due to NVT | No adult immunization policy; NVT expansion |
| Obolski, 2023 | Malawi | Observational / Genomic | Children (vaccinated/unvaccinated), HIV-infected adults | Urban community carriage surveys | PCV13 | Streptococcus pneumoniae | Whole genome sequencing of 2804 isolates (2015–2019) | Nasopharyngeal carriage isolates, WGS pipeline | Genomic AMR profiles, prevalence of resistant lineages | NR | Shift in pneumococcal population; AMR-associated NVT expansion | Observational, limited to Blantyre, Malawi |
| Kumar, 2025 | India | Review | Adults (focus on older adults with respiratory infections) | Narrative review (Indian context) | PCV13, PCV15, PCV20 (adult vaccination) | Streptococcus pneumoniae | Narrative review of burden, AMR trends, and vaccine role | Published Indian studies, national surveillance, WHO/UNICEF reports | NR | NR | Evidence of reduced hospitalizations, antibiotic prescriptions with adult PCVs | Low adult vaccination coverage; data gaps |
| Alghamdi, 2021 | Saudi Arabia (review, global relevance) | Review | NR (general focus on AMR pathogens and vaccine targets) | Narrative synthesis | Existing vaccines (PCV, Hib); candidate vaccines (C. difficile, ExPEC, S. aureus, N. gonorrhoeae, K. pneumoniae, Salmonella spp., etc.) | S. pneumoniae, H. influenzae, N. gonorrhoeae, S. aureus, K. pneumoniae, P. aeruginosa, Salmonella spp., Shigella spp., E. coli, C. difficile | Narrative review of AMR-related vaccines | Literature review (WHO, CDC, published studies) | Narrative (no primary metrics) | Narrative (discussion on reduced prescribing, bystander selection, herd effects) | Qualitative; references to reduced resistance post-PCV/Hib; potential reductions with pipeline vaccines | Vaccines under development, limited data; under-researched field; need more funding and R&D |
| Ozawa, 2021 | Ethiopia | Modeling | Children under 5 years | Country-level model (national immunization program) | PCV (13-valent) | Streptococcus pneumoniae | Agent-based modeling (DREAMR) with epidemiology, PK/PD, economics | Epidemiologic data, WHO/UNICEF immunization data, published resistance rates, PK/PD literature | Resistance prevalence, treatment failures, AMR-related mortality | DDD/1000 children per day; treatment utilization rates | PCV reduced AMR accumulation by 14.8% (amoxicillin) and 0.6% (ceftriaxone); averted ~718,100 treatment failures and 9,520 AMR deaths (2011–2017); cost savings ~$32.7m | Model assumptions; uncertainty; Ethiopia-specific; serotype replacement not fully captured |
| Chen, 2019 | Ethiopia | Modeling | Children under 5 years | Country-level model (pneumococcal disease treatment in Ethiopia) | NR (focus on antibiotic resistance impact, PCV context implicit) | Streptococcus pneumoniae | Agent-based modeling (DREAMR bacteria + human submodels) | National resistance data, WHO/UNICEF immunization estimates, literature on antibiotic use | Proportion nonsusceptible bacteria, treatment failure rate, MIC distribution | DDD; antibiotic utilization patterns (self-medication vs facility) | Estimated 195,763 treatment failures/year, 2,925 child deaths attributable to AMR, $15.8m annual cost | Uncertainty in ABM; Ethiopia-specific; herd immunity assumption; computational limits |
